# Motor competence development of children in Singapore: a cross-sectional and longitudinal study

**DOI:** 10.7717/peerj.19698

**Published:** 2025-07-31

**Authors:** Jernice S.Y. Tan, Michael Y.H. Chia

**Affiliations:** Physical Education and Sports Science, National Institute of Education, Nanyang Technological University, Singapore

**Keywords:** Movement literacy, MABC, Preschooler, Fine motor skills, Gross motor skills, Mixed study, Motor development, Southeast Asia, Physical education

## Abstract

**Introduction:**

Understanding the motor competence development of young children requires both cross-sectional and longitudinal analyses. This is crucial for identifying relative age effects and tracking individual developmental trajectories. However, there are limited data in Southeast Asia, particularly in Singapore.

**Methods:**

This study monitored the development of motor competence of 75 children in Singapore, aged 3–4.5 years, over an 18-month period. Four data points were conducted using the Movement Assessment for Children, 2nd Edition (MABC-2) at six-month intervals. Data were analyzed using repeated measures multivariate analysis of variance (MANOVA), two-way analysis of variance (ANOVA), and *post-hoc* multiple comparisons.

**Results:**

Cross-sectional analysis revealed a positive age effect across all eight motor tasks, with older children demonstrating higher fine and gross motor competence. Longitudinal analysis showed significant improvement in seven out of eight motor tasks over the 18-month period, except for the task of jumping on mats.

**Conclusion:**

The most pronounced age effect was observed between children aged 3.5 and 4.0 years, suggesting a potential period of accelerated motor development within this age range. The significant improvements in motor competence observed over the 18-month period underscore the critical nature of early childhood for motor skill acquisition. Additionally, the study highlights the importance of frequent monitoring (*e.g.*, every 6 months) to identify children with motor delays and facilitate timely interventions.

## Introduction

### Understanding motor competence—impact & multifaceted nature

Motor competence refers to the ability to perform fundamental movement skills, such as locomotor, object control, and stability, which are essential for active physical activity participation from early childhood to early adulthood (Bardid, Utesch & Lenoir, 2019). In early childhood, motor competence is considered a single latent construct supporting motor performance (Utesch et al., 2016), where high perceived competence in children drives motor skill development and physical activity. However, as children transition into middle childhood and their cognitive abilities mature, their actual motor competence becomes the primary driver, influencing their perceived competence and, consequently, their physical activity levels (Den Uil et al., 2023).

Motor competence is crucial for children’s physical health, laying the foundation for lifelong physical activity and fosters an active lifestyle from an early age (Ružbarská, 2020). Its development is influenced by growth, maturation, and environmental interactions (Malina, 2004). Systematic reviews highlight positive associations between fundamental movement skills, physical health, and physical activity (Lubans et al., 2010; Tang & Wang, 2023). While the motor competence development may occur independently of physical activity during early childhood (Schmutz et al., 2020), sedentary behavior and physical activity levels can significantly impact these developmental trajectories (Hjorth et al., 2014; Kwofie, Janssen & Reilly, 2025). This complex interplay emphasizes the role of motor competence in overall health and well-being.

Beyond physical health, many studies demonstrate relationships between motor competence and various aspects of child development including weight status, social relationships, physical activity, perceived motor competence, physical fitness, and cognitive skills (Den Uil et al., 2023; Lima et al., 2021; Herrmann et al., 2021). Loprinzi, Davis & Fu (2015) proposed that early motor competence mediates physical activity from childhood to adulthood, suggesting that boosting perceived motor competence will improve motor skill development and physical activity. Crucially, Stodden et al.’s (2008) conceptual model revealed positive relationships among perceived motor competence, actual motor competence, physical activity, and health-related fitness, particularly noting how perceived competence and activity influence motor skill development. Research also links motor competence to academic outcomes, as seen in associations between motor coordination, executive functioning, visuospatial skills, and academic success (Cameron et al., 2016). Early fine motor skills predict later visuospatial reasoning (Cortes et al., 2022), and cognitively challenging motor activities enhance executive functioning and math problem-solving (Willoughby et al., 2021). Furthermore, early gross motor skills, like walking, are associated with brain connectivity and cognitive development (Marrus et al., 2017), illustrating the broad developmental significance of motor competence.

### Investigating motor competence—trajectories & study design

Research within the past decade has expanded our understanding of motor competence development in Asia; however, significant gaps concerning longitudinal perspectives persist. From East Asia, studies such as Ke et al. (2020) in China reported significant positive age effects across all MABC-2 tasks for children aged 3-6 years. Similarly, Zhao et al. (2022) investigated fundamental motor skills in 4-5-year-old Chinese preschoolers, identifying significant relative age effects in locomotor skills and overall gross motor skills. In Southeast Asia, particularly Singapore, recent studies also provide valuable insights from a cross-sectional lens. Tan & Chia (2024) evaluated 3-6-year-old children’s motor competence using MABC-2 and observed significant positive age effects, with more pronounced differences found between 3- and 4-year-olds compared to 5- and 6-year-olds. Similarly, another Singaporean study by Tan & Lim (2025) on gross motor skills in 2.5-6-year-olds also reported positive age effects in stability, locomotion, and object manipulation, highlighting the rapid motor development in early childhood. While these recent regional studies advance our understanding of motor competence in Asian contexts, their predominantly cross-sectional design limits insights into individual developmental trajectories and the nuances of change over time.

Longitudinal studies consistently reveal the long-term impact of early-life experiences, including environmental factors, on child development. For example, Chen et al. (2023) used a short-term (1-year) longitudinal design to investigate the link between fundamental movement skills and physical fitness in preschoolers. They emphasize the immediate impact of early motor competence on physical fitness. Barnett, Salmon & Hesketh (2016)’s medium-term follow-up (19 months, 3.5, and 5 years) highlights the importance of early physical activity for motor competence by school entry. This suggests that that early engagement in physical activity is crucial for establishing a foundation for motor competence. Orri et al. (2020)’s long-term analysis of the Quebec Longitudinal Study (5 months to 21 years) demonstrates the lasting influence of early childhood factors, revealing that early experiences have profound and enduring effects on development throughout adolescence and into adulthood. Even studies focusing on cognition (*e.g.*, Brooks-Gunn, Han & Waldfogel, 2002) reinforce the interconnectedness of various developmental domains. Collectively, these studies underscore the critical role of early childhood in shaping long-term developmental trajectories and the value of sustained longitudinal monitoring.

To address the limitations of predominantly cross-sectional data and gain a more dynamic understanding of motor development, integrating both cross-sectional and longitudinal designs proves highly valuable. This combined approach offers a comprehensive strategy for monitoring the development of children’s motor competence and informing timely interventions for potential delays, by allowing for the assessment of age-related differences alongside individual developmental trajectories. For example, Longmuir, Banks & McCrindle (2012) used a cross-sectional design to assess motor performance post-heart surgery, recommending longitudinal follow-up. Understanding the developmental antecedents of motor competence and the impact of factors like sedentary behavior (Kwofie, Janssen & Reilly, 2025) and physical activity (Hjorth et al., 2014) informs interventions promoting healthy motor development. Combining these approaches, Bürgi et al. (2011) investigated the relationship between physical activity, motor skills, and health indicators in preschoolers, revealing immediate and long-term associations relevant to identifying delays and promoting healthy growth. Similarly, Niederer et al. (2011) used a combined approach to explore links between motor skills, aerobic fitness, and cognition in preschoolers, enabling assessment of immediate associations and tracking changes over time to identify early markers for cognitive development and motor proficiency. This combined approach enables the identification of potential delays and guides interventions for optimal development.

### Purpose of the study

Evidence consistently highlights the complex interplay of genetics, environment, and sociocultural factors in the development of motor competence in early childhood (Chen et al., 2023; Herrmann et al., 2021; Willoughby et al., 2021). Previous regional studies are largely cross-sectional or limited in longitudinal scope (Tan & Chia, 2024; Tan & Lim, 2025), the present study complements the knowledge base by providing a nuanced understanding of children’s developmental changes in motor competence. We provide the first comprehensive, combined cross-sectional and longitudinal investigation of motor competence in young children in Singapore. By measuring developmental trajectories at six-month intervals over an 18-month period, we offer a detailed examination of normative motor developmental patterns, individual variations, and relative age effects specifically within a unique Southeast Asian context. This detailed, longitudinal perspective from a previously under-researched population enriches the global understanding of motor development and establish crucial baseline data for targeted physical health initiatives, future interventions, and policymaking in Singapore and the broader Southeast Asian region with similar diverse populations.

## Methods

### Research model

This study adopted a combined cross-sectional and longitudinal research design. At baseline (P1; see Table 1), children from different age groups were recruited and assessed, constituting the cross-sectional component of the dataset. Subsequently, all participants were followed longitudinally, undergoing repeated tests of their motor competence at six-month intervals over an 18-month period. This methodology enabled the examination of both age-related differences and individual developmental trajectories over time within the study population.

**Table 1:** Number of children in each age and gender group.

Age (years)	Female	Male	Overall
3.0	17	19	36
3.5	5	10	15
4.0	11	6	17
4.5	2	5	7
Total	35	40	75

**Notes.**

Children were assigned to half-year age groups: 3.0 years: 3 years to <3.5 years; 3.5 years: 3.5 years to <4 years; 4.0 years: 4 years to <4.5 years; 4.5 years: 4.5 years to <5 years.

### Participants

A total of 75 children (40 boys, 35 girls) with a mean age of 3.5 ± 0.5 years old were recruited at baseline (P1; see Table 1). They were categorized into half-year age increments (*e.g.*, 3.0 years: 3 years to <3.5 years; 4.5 years: 4.5 years to <5 years) based on their age range at P1. This categorization aligned with MABC-2 normative data (6-month ranges, 3:0-4:11) and facilitated our study’s aim of six-monthly motor competence analysis. Participants were reported as healthy without known medical conditions or disabilities. Sample size was determined *via* G*Power statistical analysis (Faul et al., 2007). With a statistical significance level of 0.05, a medium effect size (*η*_p_^2^ = 0.06), and a statistical power of 0.9, a minimum of 64 participants was recommended for the mixed-design repeated measures multivariate analysis of variance (MANOVA). Ethical approval for the study was secured from the institutional review board (Republic Polytechnic, Singapore; IRB reference code: SHL-M-2014-016). Prior to data collection, written informed parental consent and child assent were obtained.

### Instruments & tasks

The Movement Assessment Battery for Children, 2nd Edition (MABC-2; Henderson, Sugden & Barnett, 2007) is a widely validated assessment tool used in research related to the motor development of children aged 3-16 years. Its simplicity and short administration time make it a popular choice for identifying motor delays and guiding interventions (Hadwin et al., 2023). This study utilized only the MABC-2 tasks designed for age band 1 (3–6 years old). The MABC-2 consists of eight tasks categorized into three components: Manual dexterity: MD1–posting coins, MD2–threading beads, MD3–drawing trails; Aiming & catching: AC1–catching beanbags, AC2–throwing beanbags onto target; Balance: BAL1–one-leg balance, BAL2–walking heels raised, BAL3–jumping on mats.

### Procedures

**Figure 1:**
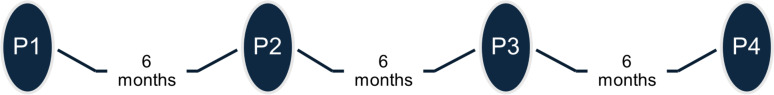
Timeline for the data collection phases.

The study was conducted indoors, beginning with a circle time activity to familiarize children with the testers and their environment. To mitigate novelty and fatigue influences, test sequences were randomized. Participants were allowed to wear or remove footwear, as footwear use was found to have no significant impact on stability and locomotion tasks (Tan, 2019). Trained testers provided standardized instructions and demonstrations. To ensure reliability, testers underwent familiarization training and practiced with reliability videos, achieving high intra- and inter-tester reliability (intra-class coefficient index > 0.7; (Shrout & Fleiss, 1979). Each participant received one practice attempt and up to two actual trials for each test, with standardized instructions and demonstrations provided by the testers. Each testing session lasted approximately 20 min. Data collection occurred in four test phases, spaced six months apart: baseline (P1), 6 months post-baseline (P2), 12 months post-baseline (P3) and 18 months post-baseline (P4; see Fig. 1).

### Data reduction & analysis

Raw scores (RS) from the best actual trial of each MABC-2 task were recorded for each participant, resulting in an initial eight RS, except in cases of failure or refusal. For tasks MD1 and BAL1, the RS for the preferred/non-preferred hand and best/other leg were averaged respectively to obtain the final eight RS. For MD tasks, lower scores indicate better motor competence, while the opposite is true for AC and BAL tasks, where higher scores represent better performance. The MD task protocols differed by age group (3–4 *vs.* 5–6 years old). Participants completed the MD1 and MD2 tasks with six coins and six beads, respectively, for younger participants, and 12 coins and 12 beads for older participants. To ensure comparability across all four test phases (P1, P2, P3 & P4), the raw scores (RS) for MD1 and MD2 were doubled for participants younger than 5 years old during data analysis. This approach, similar to Chow, Henderson & Barnett (2001), allowed for a more accurate comparison of developmental changes. A mixed-design repeated measures MANOVA, followed by two-way ANOVA tests was used to identify significant differences across the eight dependent variables by age and test phase at *p* < .05. For *post-hoc* multiple comparisons, the Holm-Bonferroni sequential correction was applied to control the family-wise error rate such that subsequent *p* value comparison will be conducted if earlier *p* value is significant. This robust step-down procedure involves sorting the unadjusted *p*-values from smallest to largest and comparing each *p*_(i)_ against a critical alpha level of α/(k−i+1), where k is the total number of comparisons in the family and i is the rank of the *p*-value. For a family of three comparisons (*e.g.*, P1 *vs* P2; P2 *vs* P3; P3 *vs* P4) at α = .05, the *p*-values would be sequentially compared against α/3 = .0167, α/2 = .025, and α/1 = .05, respectively. This ensures that the probability of making at least one Type I error across the family of comparisons remains below .05. Effect sizes (*η*_p_^2^) were calculated and interpreted using Cohen’s (1988) criteria: 0.01 (small), 0.06 (medium), and 0.14 (large). All statistical analyses were conducted using SPSS Version 28 (IBM Corp., Armonk, NY, USA).

## Results

Repeated-measures MANOVA tests were conducted on the raw scores (RS) of eight individual motor tasks. The cross-sectional analysis by age used only RS at baseline (P1). The longitudinal analysis by phase used RS at P1, P2, P3, and P4, with a 6-month interval between phases. The MANOVA results revealed significant age differences between subjects across the eight tasks (F(24,186) = 3.124, *p* = .001, Pillai’s Trace = 0.862, *η*_p_^2^= 0.287). This indicates a large effect size, as 28.7% of the variance in motor competence is explained by age group. Additionally, significant within-subject differences were observed across the four test phases for all eight tasks (F(24,44) = 10.007, *p* = .001, Pillai’s Trace = 0.845, partial *η*_p_^22^ = 0.845). This represents a very large effect size, where 84.5% of the variance in motor competence is attributable to the change in test phase. An overall interaction effect between age and phase was also found (F(72,138) = 1.473, *p* = .027, Pillai’s Trace = 1.303, partial *η*_p_^2^ = 0.434), indicating a large effect size where 43.4% of the variance is explained by the interaction of age and test phase.

**Table 2:** Results of children (*n* = 75) across phases by age groups of eight individual tasks.

	P1	P2	P3	P4	Phase	Age	Phase × Age
					*F*		
	*Mean (SD)*				*p* value		
					Effect size (*η*_p_^2^)		
MD1: Posting coins							
Age 3.0	30.94 (6.15)	28.17 (3.95)	25.89 (3.58)	23.56 (6.54)	20.32	12.28	2.29
Age 3.5	33.73 (14.91)	27.60 (6.85)	24.27 (3.70)	22.33 (3.27)	(.001*)	(.001*)	(.032*)
Age 4.0	24.24 (3.07)	23.06 (2.75)	22.41 (1.81)	20.06 (2.93)	0.233	0.355	0.093
Age 4.5	23.71 (5.82)	20.14 (1.87)	21.43 (3.60)	18.71 (2.22)			
MD2: Threading beads							
Age 3.0	105.3 (32.86)	92.72 (22.85)	77.50 (17.05)	64.00 (20.06)	30.94	13.77	0.89
Age 3.5	90.93 (34.33)	82.00 (25.06)	65.33 (14.93)	57.07 (16.95)	(.001*)	(.001*)	(.520)
Age 4.0	68.71 (16.34)	65.12 (16.50)	50.82 (9.86)	45.71 (6.62)	0.316	0.381	0.038
Age 4.5	66.86 (7.01)	58.71 (11.31)	53.00 (8.17)	43.43 (6.40)			
MD3: Drawing trail							
Age 3.0	8.42 (4.90)	4.56 (3.63)	2.58 (2.62)	1.11 (1.17)	18.41	12.34	5.02
Age 3.5	5.93 (4.46)	3.40 (3.02)	3.07 (3.39)	1.13 (1.60)	(.001*)	(.001*)	(.001*)
Age 4.0	2.41 (2.09)	0.94 (1.03)	0.82 (1.02)	0.65 (0.70)	0.216	0.356	0.184
Age 4.5	1.14 (0.90)	0.57 (1.13)	1.29 (1.80)	0.14 (0.38)			
AC1: Catching beanbags							
Age 3.0	1.42 (1.52)	2.61 (2.17)	3.89 (2.24)	5.17 (2.30)	10.69	11.11	2.33
Age 3.5	3.93 (2.79)	3.87 (2.13)	4.67 (3.20)	6.07 (2.38)	(.001*)	(.001*)	(.016*)
Age 4.0	5.06 (2.59)	4.88 (2.03)	5.00 (2.09)	6.41 (1.62)	0.138	0.332	0.094
Age 4.5	5.43 (1.13)	4.71 (2.75)	6.71 (2.43)	6.29 (1.50)			
AC2: Throwing beanbags onto mat							
Age 3.0	2.33 (1.94)	2.61 (1.81)	3.31 (1.37)	4.17 (1.68)	8.17	6.80	1.54
Age 3.5	2.60 (2.29)	3.20 (2.18)	3.73 (1.62)	5.00 (1.96)	(.001*)	(.001*)	(.143)
Age 4.0	3.82 (2.01)	4.12 (1.65)	3.53 (1.38)	4.47 (1.88)	0.109	0.234	0.064
Age 4.5	3.43 (2.15)	5.43 (1.62)	4.86 (1.68)	5.29 (1.50)			
BAL1: One-leg balance							
Age 3.0	6.22 (4.06)	6.972 (4.70)	9.000 (7.01)	14.86 (10.37)	30.12	11.16	1.42
Age 3.5	6.67 (6.07)	5.533 (3.70)	10.67 (8.22)	15.73 (9.45)	(.001*)	(.001*)	(.197)
Age 4.0	11.24 (6.47)	15.24 (7.89)	18.71 (9.14)	21.18 (8.95)	0.310	0.333	0.060
Age 4.5	14.00 (9.18)	18.57 (7.74)	22.00 (8.35)	27.71 (5.62)			
BAL2: Walking heels raised							
Age 3.0	9.28 (5.56)	11.22 (4.11)	11.72 (4.44)	14.47 (2.21)	9.77	4.50	1.77
Age 3.5	7.67 (6.46)	10.67 (5.25)	11.60 (5.96)	15.00 (0.00)	(.001*)	(.006*)	(.083)
Age 4.0	12.53 (4.35)	13.29 (3.53)	15.00 (0.00)	14.18 (2.92)	0.127	0.168	0.073
Age 4.5	14.29 (1.89)	14.29 (1.89)	11.43 (5.65)	15.00 (0.00)			
BAL3: Jumping on mats							
Age 3.0	4.42 (1.13)	4.58 (0.97)	4.42 (1.08)	4.86 (0.54)	1.81	3.46	0.76
Age 3.5	4.07 (1.67)	4.27 (1.44)	4.07 (1.62)	4.87 (0.52)	(.149)	(.021*)	(.648)
Age 4.0	4.82 (0.39)	4.88 (0.33)	4.82 (0.73)	4.77 (0.97)	0.026	0.134	0.033
Age 4.5	5.00 (0.00)	5.00 (0.00)	4.86 (0.38)	5.00 (0.00)			

**Notes.**

MD1: average of preferred MD1 & non-preferred MD1 completion time in secs (six coins for 3 & 4 years; 12 coins for 5 & 6 years); MD2: completion time in secs (six beads for 3 & 4 yrs; 12 beads for 5 & 6 years); As 3 & 4-year-olds complete six instead of 12 coins & beads, the scores for these 2 age groups were multiplied by 2 for comparative analysis with 5 & 6-year-olds. MD3: n. of errors; AC1: n. of correct catches out of 10 (by hands or by trapping ball against body for 3 & 4 years; only by hands for 5 & 6 years); AC2: n. of correct throws out of 10; BAL1: average of best & other leg balance time, up to 30 secs; BAL2: n. of correct steps up to 15; BAL3: n. of correct jumps up to 5 (discontinuous jumps, feet can adjust & feet not landing together allowed for 3 & 4 years; continuous jumps, feet cannot adjust & both feet must land together for 5 & 6 years). (*) MANOVA analysis showed significant results at *p* < .05.

The follow-up two-way ANOVA test within subjects revealed significant differences across phases for seven out of eight tasks, with the exception of the BAL3 task (see Table 2). These significant within-subject effects indicate that changes in motor competence over time are evident for most tasks. Conversely, the between-subjects ANOVA test showed significant age differences for all eight tasks (see Table 2), with effect sizes ranging from medium to large (*η*_p_^2^ ≥ 0.06). This suggests that age group accounts for a meaningful proportion of the variance in motor performance across all tasks, indicating practically important differences in skill levels between different age cohorts. For interaction effects (phase × age), three out of eight tasks (MD1, MD3 & AC1) exhibited significant results with medium to large effect size (see Table 2), with effect sizes also ranging from medium to large (*η*_p_^2^ ≥ 0.06). This implies that the developmental trajectories of these specific motor tasks differ considerably depending on the child’s age, highlighting age-specific patterns of change in motor competence.

Multiple comparisons revealed age-related motor competence. Significant improvements were observed between 3.0 to 3.5 years old (AC1 only), and more broadly from 3.5 to 4.0 years old (six out of eight tasks). Notably, no significant changes between 4.0 to 4.5 years old (see Table 3), suggesting a stabilization of motor development in this older group. Phase comparisons showed progressive improvements within subjects over time. Initial significant gains were seen from P1–P2 (five out of eight tasks) and P2–P3 (four out of eight tasks). A particularly notable significant increase was evident between P3–P4 (six out of eight tasks; see Table 3). Interestingly, Task-specific analyses highlighted varied developmental trajectories. Three tasks (MD1, MD2 & BAL1) consistently improved across all phases, indicating sustained development. Conversely, MD3 and AC2 showed improvements in only two phases, while AC1 and BAL2 exhibited minimal change, suggesting slower or earlier stabilization for these particular tasks and finally BAL3 exhibited non-significant change at all (see Table 3).

**Table 3:** *Post-hoc* tests using multiple comparisons by age and phase.

Tasks	MD1	MD2	MD3	AC1	AC2	BAL1	BAL2	BAL3
	3.0 *vs* 3.5 years old							
SE	1.132	4.925	0.621	0.473	0.323	1.794	0.841	0.205
*p*	.630	.081	.498	.007^*^	.251	.673	.256	.051
	3.5 *vs* 4.0 years old							
SE	1.318	5.733	0.723	0.550	0.376	2.089	0.979	0.238
*p*	.001^*^	.003^*^	.001^*^	.187	.108	.003^*^	.003^*^	.006^*^
	4.0 *vs* 4.5 years old							
SE	1.727	7.513	0.947	0.721	0.492	2.738	1.283	0.312
*p*	.441	.765	.609	.292	.152	.125	.873	.656
								
	P1 *vs* P2							
SE	1.150	4.089	0.565	0.392	0.382	0.949	0.806	0.172
*p*	.001^*^	.048^*^	.001^*^	.950	.019^*^	.021^*^	.051	.572
	P2 *vs* P3							
SE	0.661	3.353	0.419	0.405	0.333	1.027	0.800	0.174
*p*	.016^*^	.001^*^	.324	.010^*^	.093	.002^*^	.634	.565
	P3 *vs* P4							
SE	0.751	2.299	0.380	0.401	0.315	1.115	0.661	0.169
*p*	.012^*^	.001^*^	.004^*^	.045	.014^*^	.001^*^	.002^*^	.043

**Notes.**

Manual dexterity: MD1–posting coins, MD2–threading beads, MD3–drawing trails; Aiming & catching: AC1–catching beanbags, AC2–throwing beanbags onto target; Balance: BAL1–one-leg balance, BAL2–walking heels raised, BAL3–jumping on mats. *Post-hoc* tests showed significant results with * at smallest *p* < .05/3 = .0167 to next smaller *p* < .05/2 = 0.25 (if smallest *p* is significant) and to largest *p* (if next smaller *p* is significant) and to *p* < .05/1 = .05 (if 2 earlier *p* are significant) for respective age and phase comparison.

## Discussion

This study examined the cross-sectional and longitudinal changes in the development of motor competence of young children in Singapore. Children aged 3.0 to 4.5 years were followed up for 18 months. Motor competence was measured four times, separated by six-month intervals. Children were aged between 4.5 and 6.0 years by the end of this study. Significant age-related differences in motor competence were observed, as indicated by improved performance with increasing age in all eight tasks. Of the eight tasks, seven demonstrated significant improvements across the four test phases

### Children’s motor competence—a cross-sectional perspective

Multiple studies confirm that age positively influences motor development, particularly between ages 2 and 7 years (Ke et al., 2020; Tang & Wang, 2023), with biological maturation being the key to achieving motor milestones (Malina, 2004; Lubans et al., 2010). For example, Tan & Chia (2024) observed positive annual age effects in eight out of eight tasks for 3- to 4-year-olds and six out of eight tasks for 4- to 5-year-olds, indicating continuous motor development in these age groups. However, the relative age effect is also noted, where children born earlier in the year perform better (Navarro-Patón, 2021; Zhao et al., 2022). Furthermore, Tan & Lim (2025) observed that stability and object manipulation skills in Singaporean children showed significant annual differences throughout early and middle childhood. Significant annual differences in locomotion were noted in early developmental years. Despite this, a half-yearly analysis indicated that most locomotion tasks, with the exception of hopping, progressed significantly in early childhood but then showed non-significant improvements later. They concluded that skills requiring greater postural control and torso strength develop more significantly in middle and later childhood, whereas simpler locomotion tasks see their most marked progress in early childhood.

In the present study, our findings of significant positive age effects between 3.0 and 4.0 years, particularly between 3.5 and 4.0 years, indicate a period of rapid motor development. This suggests that even minor age differences (six months) can result in significant disparities in motor competence, with older children demonstrating better performance. Conversely, the lack of significant age effects between 4.0 and 4.5 years suggests a stabilization of motor skills in this older age range, implying that children within this specific group could be collectively targeted for motor programs. This distinct pattern contrasts with the broader 3.0–4.5-year range, which should not be grouped uniformly. Indeed, our study showed that only three tasks (MD1, MD2 & BAL1) consistently improved throughout all the phases, underscoring that not all motor skills follow the same developmental trajectory. Although this was an observational study that did not evaluate interventions, our findings advocate for regular follow-up every six months in cases where motor delay persists beyond one year of age and the early institution of intervention when improvement is not observed.

### Children’s motor competence—a longitudinal perspective

This study employed a more frequent motor assessment schedule, with testing conducted every six months, offering a more granular view of motor development than typical longitudinal studies (Barnett, Salmon & Hesketh, 2016; Bürgi et al., 2011; Chen et al., 2023; Orri et al., 2020). Motor competence in all tasks showed significant improvements a few times during the 18-month period, with the exception of one task (BAL3: jumping on mats). These findings support the notion that early childhood, ages 2–7 years, is a ‘golden period’ for motor development (Chen et al., 2023; Figueroa & An, 2017). This period is also a sensitive learning window, characterized by rapid acquisition of motor skills. As children reach specific ages, their motor skills improve in stages to meet different developmental milestones. The results suggest that six-month intervals could be effective for capturing motor developmental changes. However, the task protocol of jumping on mats (BAL3) may not be a reliable measure of motor skill improvement in this age group.

The lack of significant improvement in BAL3 could be explained when we observed that at baseline, children’s scores averaged above 4.0 out of 5.0. With a limited 0–5 scale and only six discrete points, detecting statistically significant differences becomes challenging. This aligns with Tan & Lim’s (2025) findings, where non-significant differences in consecutive horizontal jumping were reported over a six-month age difference when scores ranged from 3.8 to 4.8 out of 5.0 (ages 4.0–6.0). Consequently, using raw scores with a limited range for tasks like consecutive jumping over mats may not be optimal for assessing motor skill improvement. Standardizing raw scores through normalization could offer a more sensitive measure of statistical differences. Alternatively, increasing the difficulty of the jumping task could enhance its ability to differentiate motor competence between children of similar ages.

### Strengths and limitations

A key strength of this study was its rigorous methodology, which utilized the MABC-2—a widely used and highly reliable tool for assessing motor competence. This enabled the identification of developmental windows for intervention, revealing a critical period of accelerated motor gains in young children through longitudinal tracking. This level of detail offers vital insights for targeted strategies, addressing a significant gap in the current literature that often uses broader age groupings. By effectively bridging both cross-sectional and longitudinal evidence, the study provided a nuanced understanding of motor development, demonstrating heterogeneous developmental trajectories where not all motor competence improves uniformly. These combined strengths offer critical, actionable insights for practitioners, policymakers, and researchers, guiding both intervention strategies and assessment practices.

However, the study has its limitations. The exclusion of children with known medical conditions may limit the generalizability of findings to broader populations. Furthermore, the standardized testing environment might not fully capture children’s natural motor competence, and testing conditions could not always account for their emotional readiness. Specifically, varying preschool schedules meant some children were tested before lunch or after naptime, which can affect cooperation and focus, particularly in younger participants. Future research should strive for more standardized testing times to minimize these influences. Additionally, adopting more inclusive recruitment and naturalistic assessment methods could enhance generalizability and ecological validity. Addressing the logistical challenges inherent in longitudinal studies will also be crucial for continued progress in this field.

## Conclusion

This study examined cross-sectional and longitudinal developmental trajectories of motor competence in young children in Singapore. The cross-sectional analysis showed an age and relative age positive effect on motor competence, and the longitudinal analysis indicated significant motor skill improvement over 18 months. The MABC-2 was sensitive to charting improvements over six-monthly periods, suggesting that even shorter intervals may be appropriate when monitoring intervention programs. For children with motor development delays exceeding one year period, early evaluation by healthcare professionals is recommended. Overall, semi-annual observations provide valuable insights for monitoring motor development trends in young children.

## Supplemental Information

10.7717/peerj.19698/supp-1 Supplemental Information 1 Dataset

10.7717/peerj.19698/supp-2 Supplemental Information 2 Codebook

## Additional Information and Declarations

### Data Availability

The following information was supplied regarding data availability: The dataset shows the raw scores of 75 child participants collected over 4 data points using MABC-2.
